# RACK1 promotes autophagy via the PERK signaling pathway to protect against traumatic brain injury in rats

**DOI:** 10.1111/cns.14691

**Published:** 2024-03-26

**Authors:** Haibo Ni, Xugang Kan, Qin Rui, Yang Zhang, Weiwei Zhai, Baole Zhang, Zhengquan Yu

**Affiliations:** ^1^ Department of Neurosurgery & Brain and Nerve Research Laboratory The First Affiliated Hospital of Soochow University Suzhou China; ^2^ Department of Neurosurgery The Fourth Affiliated Hospital of Soochow University Suzhou China; ^3^ Department of Neurobiology and Cell Biology, Xuzhou Key Laboratory of Neurobiology Xuzhou Medical University Xuzhou China; ^4^ Department of Center of Clinical Laboratory The Fourth Affiliated Hospital of Soochow University Suzhou China

**Keywords:** autophagy, neuroprotection, PERK, RACK1, traumatic brain injury

## Abstract

**Aims:**

Neuronal cell death is a primary factor that determines the outcome after traumatic brain injury (TBI). We previously revealed the importance of receptor for activated C kinase (RACK1), a multifunctional scaffold protein, in maintaining neuronal survival after TBI, but the specific mechanism remains unclear. The aim of this study was to explore the mechanism underlying RACK1‐mediated neuroprotection in TBI.

**Methods:**

TBI model was established using controlled cortical impact injury in Sprague–Dawley rats. Genetic intervention and pharmacological inhibition of RACK1 and PERK‐autophagy signaling were administrated by intracerebroventricular injection. Western blotting, coimmunoprecipitation, transmission electron microscopy, real‐time PCR, immunofluorescence, TUNEL staining, Nissl staining, neurobehavioral tests, and contusion volume assessment were performed.

**Results:**

Endogenous RACK1 was upregulated and correlated with autophagy induction after TBI. RACK1 knockdown markedly inhibited TBI‐induced autophagy, whereas RACK1 overexpression exerted the opposite effects. Moreover, RACK1 overexpression ameliorated neuronal apoptosis, neurological deficits, and cortical tissue loss after TBI, and these effects were abrogated by the autophagy inhibitor 3‐methyladenine or siRNAs targeting Beclin1 and Atg5. Mechanistically, RACK1 interacted with PERK and activated PERK signaling. Pharmacological and genetic inhibition of the PERK pathway abolished RACK1‐induced autophagy after TBI.

**Conclusion:**

Our findings indicate that RACK1 protected against TBI‐induced neuronal damage partly through autophagy induction by regulating the PERK signaling pathway.

## Graphical abstract

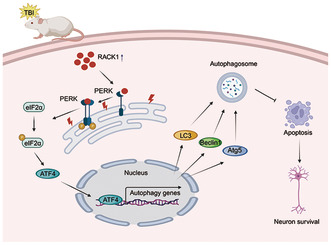

## 1 INTRODUCTION

Traumatic brain injury (TBI) is a major public health issue and a leading cause of mortality and disability worldwide, and it is followed by long‐term physical and cognitive consequences.^1^ The pathogenesis of TBI is highly complex, involving both primary injury, which is caused by the initial biomechanical effects of force on brain tissues, and subsequent secondary injury, which is caused by the activation of multiple pathophysiological cascades.^2^ Neuronal cell death is one of the most prominent pathological features of secondary injury, and it is a deciding factor in determining the outcome of patients with TBI.^3^ Therefore, alleviating neuronal apoptosis and promoting endogenous neural repair after TBI are promising therapeutic strategies for improving the prognosis of TBI.

Autophagy is a conserved intracellular catabolic mechanism that facilitates the degradation of aberrant proteins and damaged organelles in the cytoplasm via the autophagosomal‐lysosomal pathway, and it is crucial for maintaining cellular homeostasis and regulating cell survival.^4^ In the early phase of TBI, autophagy is induced and plays dual roles, both beneficial and detrimental roles, in neuronal cell death.^5^, ^6^ Accumulating evidence indicates that endoplasmic reticulum (ER) stress plays a crucial role in modulating autophagy.^7^ As a master transmembrane ER stress sensor, protein kinase RNA‐like ER kinase (PERK) participates in the autophagy process via the activating transcription factor 4 (ATF4)‐mediated transcriptional upregulation of components of autophagy machinery.^8^ The induction of autophagy via PERK pathway activation by several drugs, such as melatonin and gelsenicine, has recently been shown to promote neuronal survival and functional recovery after ischemic stroke.^9^, ^10^ However, no reports have examined the role of PERK‐autophagy signaling in TBI.

Receptor for activated C kinase 1 (RACK1) is a multifaceted scaffold protein with seven‐repeat tryptophan‐aspartic acid (WD) domains.^11^ The structural characteristics of RACK1 enable it to interact with multiple signaling proteins and perform a variety of regulatory functions in multiple cells.^12^ In the brain, RACK1 is mainly localized in neurons, and its aberrant expression has been shown to be involved in the pathogenesis of several neurological disorders, including ischemic stroke,^13^ Alzheimer's disease,^14^ and Parkinson's disease.^15^ Our previous study demonstrated that RACK1 expression was increased in neurons and contributed to protecting neurons against apoptosis after TBI in a rat brain contusion model.^16^ Recently, RACK1 has been proposed to be a positive regulator of autophagic activity, and it is beneficial for cell survival and the maintenance of conditioned place preference memory.^17^, ^18^ However, whether RACK1 regulates autophagy during TBI and whether the regulatory effect of RACK1 on autophagy contributes to its neuroprotective function are unclear.

In this study, we aimed to elucidate the mechanism underlying RACK1‐mediated neuroprotection after TBI and to elucidate the role of autophagy in this process. To illustrate these points, this study was designed to explore (i) the effects of RACK1 on autophagic activity during TBI; (ii) whether RACK1 exerts its neuroprotective effects against TBI in an autophagy‐dependent manner; and (iii) the involvement of PERK signaling in RACK1‐regulated autophagy after TBI.

## 2 MATERIALS AND METHODS

### 2.1 Experimental animals

Adult male Sprague–Dawley rats (280–300 g) were purchased from the Animal Center of the Chinese Academy of Sciences (Shanghai, China). The rats were housed under controlled conditions, including 12‐h light/dark cycles and regulated temperature and humidity, and the rats were given free access to food and water. All the experimental protocols were approved by the Animal Care and Use Committee of Soochow University and conducted in accordance with the Animal Research: Reporting of In Vivo Experiments (ARRIVE) Guidelines.

### 2.2 Traumatic brain injury model establishment

Animals were randomly selected and subjected to controlled cortical impact (CCI) to establish a TBI model, as previously described.^19^ Briefly, the rats were anesthetized with isoflurane (4% for induction, 3% in oxygen for maintenance at a flow rate of 500–700 mL/min) and positioned in a stereotaxic frame. A midline incision was made to expose the skull, and a 4.5 mm‐diameter craniotomy was performed on the right parietal bone midway between the lambda and bregma, leaving the dura mater intact. Then, moderate CCI brain injury was induced using a Precision Impactor Device 68099II (RWD Life Science, Shenzhen, China) with a 4 mm‐diameter tip. The impact was delivered with the following parameters: a unilateral depth of 2.5 mm, a velocity of 4 m/s, and a dwell time of 0.25 s. After injury, the incision was closed with wound clips. Body temperature was monitored and maintained at 37°C throughout the surgical procedure with a thermostatically controlled heating pad. Sham‐operated animals received craniotomy but no impact from the CCI device.

### 2.3 Intracerebroventricular injection

Intracerebroventricular drug administration was performed as previously described.^16^ Briefly, anesthetized rats were fixed in a stereotaxic apparatus. Through a burr hole on the skull, a 10 μL Hamilton syringe needle was inserted into the right lateral ventricle at coordinates of 1.5 mm posterior, 1.0 mm lateral, and 3.5 mm below the horizontal plane of the skull. According to the manufacturer's instructions, a total volume of 5 μL (500 pmol) of siRNA or plasmid mixed with in vivo transfection reagent (Engreen, China) was injected at a rate of 0.5 μL/min 24 h before CCI. At 72 h after the injection, the rats were sacrificed and the pericontusional cortex was extracted for proteins and mRNAs. The autophagy inhibitor 3‐methyladenine (3‐MA, 400 and 600 nmol, MedChemExpress, USA)^20^, ^21^ and the PERK inhibitor GSK2606414 (90 μg/μL, Selleck Chemicals, USA)^22^ were dissolved in 0.5% DMSO, and then 5 μL was injected into the right lateral ventricle 1 h before CCI. An equivalent volume of 0.5% DMSO (5 μL) was administered to the vehicle group. The needle was left in place for an additional 10 min and then withdrawn slowly over 5 min to avert leakage. The burr hole was sealed with bone wax, and the skin incision was closed with sutures. siRNAs targeting rat RACK1, Beclin1, Atg5, and ATF4, as well as scrambled siRNA, were synthesized by Ribo Biotechnology Co., Ltd. (Guangzhou, China). To ensure knockdown efficiency, the siRNA used in this study was a pool of three different siRNA duplexes (Table 1). The RACK1 plasmid and empty vector were constructed by Genescript (Nanjing, China) as described in our previous publication.^16^

**TABLE 1:** Details of siRNAs used in this work.

Name	Target sequence (5′–3′)	
siRACK1‐1#	Sense: GGATGAGAGTCATTCAGAA	Antisense: TTCTGAATGACTCTCATCC
siRACK1‐2#	Sense: CCACTTTGTTAGCGATGTT	Antisense: AACATCGCTAACAAAGTGG
siRACK1‐3#	Sense: GCAAGCACCTTTACACATT	Antisense: AATGTGTAAAGGTGCTTGC
siAtg5‐1#	Sense: GACGGATTCCAACGTGCTT	Antisense: AAGCACGTTGGAATCCGTC
siAtg5‐2#	Sense: GAAGGTTATGAGACAAGAA	Antisense: TTCTTGTCTCATAACCTTC
siAtg5‐3#	Sense: GAGGCTCACTTTATGTCAT	Antisense: ATGACATAAAGTGAGCCTC
siBeclin1‐1#	Sense: CAATAAGATGGGTCTGAAA	Antisense: TTTCAGACCCATCTTATTG
siBeclin1‐2#	Sense: GCTCAGTACCAGCGAGAAT	Antisense: ATTCTCGCTGGTACTCAGC
siBeclin1‐3#	Sense: GCCAGACAGTGTTGTTGCT	Antisense: AGCAACAACACTGTCTGGC
siATF4‐1#	Sense: GCCACGTTGGATGACACAT	Antisense: ATGTGTCATCCAACGTGGC
siATF4‐2#	Sense: GGAAGTGAGGTTGATATCT	Antisense: AGATATCAACCTCACTTCC
siATF4‐3#	Sense: CCTCACTGGCGAGTGTAAA	Antisense: TTTACACTCGCCAGTGAGG

### 2.4 Tissue processing

At the indicated time points post‐CCI, the rats were euthanized with isoflurane and transcardially perfused with saline. To isolate proteins and mRNAs, the cortical cortex located within 3 mm of the contusion site margin (or an anatomically matched cortical area on sham rats) was collected and immediately stored at −80°C until use. To prepare frozen sections, the rats were transcardially perfused with saline followed by 4% paraformaldehyde. After adequate perfusion, the brains were extracted, postfixed in 4% paraformaldehyde overnight, and then immersed in 30% sucrose until they sank to the bottom. Serial coronal slices (10‐μm thick) were prepared using a cryostat (Leica, Germany) and mounted onto glass slides. To prepare paraffin sections, postfixed brains were embedded in paraffin. Coronal sections (5‐μm thick) were serially cut and mounted on glass slides for further staining.

### 2.5 Western blotting analysis

Tissue samples from the pericontusional cortex or corresponding ipsilateral cortex were homogenized in RIPA buffer containing phosphatase inhibitors (Beyotime, China). After centrifugation at 13,000 *g* for 30 min at 4°C, the supernatants were collected, and the protein concentrations were measured using the bicinchoninic acid (BCA) method with a Pierce™ BCA Protein Assay Kit (Thermo Fisher, USA). Equal amounts of protein were loaded onto SDS–PAGE gels (Yeasen, China) and transferred to polyvinylidene difluoride (PVDF) membranes (Millipore, USA). Subsequently, the membranes were blocked with 5% BSA blocking buffer (Beyotime, China) for 1 h at room temperature and incubated overnight at 4°C with the following antibodies: mouse anti‐RACK1 (1:200; Santa, sc‐17754, USA), rabbit anti‐LC3 (1:3000; Abcam, ab48394, UK), rabbit anti‐SQSTM1 (1:1000; Abcam, ab109012, UK), rabbit anti‐Beclin1 (1:1000; Abcam, ab210498, UK), rabbit anti‐Atg5 (1:1000; Cell Signaling, 12994S, USA), rabbit anti‐PERK (1:1000; Cell Signaling, 3192S, USA), rabbit anti‐phospho‐PERK (1:1000; Cell Signaling, 3179S, USA), rabbit anti‐eIF2α (1:1000; Cell Signaling, 9722S, USA), rabbit anti‐phospho‐eIF2α (1:1000; Cell Signaling, 9721S, USA), rabbit anti‐ATF4 (1:1000; Cell Signaling, 11815S, USA), rabbit anti‐Bcl‐2 (1:1000; Abcam, ab59348, UK), rabbit anti‐Bax (1:1000; Proteintech, 50599‐2‐Ig, China), and mouse anti‐β‐actin (1:10,000, Cell Signaling, 3700S, USA) antibodies. The membranes were then incubated in solution containing HRP‐conjugated secondary antibodies for 2 h at room temperature, and protein bands were visualized using an ECL Plus Chemiluminescence kit (Amersham Biosciences, USA). The relative densities of the target protein bands were analyzed using ImageJ software (NIH, USA).

### 2.6 Transmission electron microscopy (TEM)

TEM was utilized to examine the morphology and quantity of autophagosomes in neurons 48 h post‐CCI. Briefly, rats were transcardially perfused with ice‐cold saline followed by 4% paraformaldehyde and 2.5% glutaraldehyde. Pericontusional cortex tissues were dissected into 1 mm^3^ specimens and then fixed in 2.5% glutaraldehyde for 24 h, followed by incubation in 1% osmium tetroxide for 1 h. After dehydration in acetone solutions, the tissues were embedded in epoxy resin. Then, ultrathin sections (70 nm) were collected on copper grids, stained with uranyl acetate and lead citrate, and observed by an investigator who was blinded to the experimental groups under an electron microscope.

### 2.7 Immunofluorescence staining

Double‐fluorescence staining was performed as previously described.^23^ Frozen sections (10‐μm thick) were rinsed 3 times for 30 min with 0.1% Triton X‐100 and then blocked for 1 h with 5% goat serum (Beyotime, China). Then, the brain sections were incubated overnight at 4°C with primary antibodies, including mouse anti‐RACK1 (1:80; Santa, sc‐17754, USA), rabbit anti‐LC3 (1:200; Proteintech, 14600‐1‐AP, China), rabbit anti‐PERK (1:200; Cell Signaling, 3192S, USA), and mouse anti‐NeuN (1:200; EMD Millipore, MAB377, USA) antibodies. Next, appropriate fluorescence dye‐conjugated secondary antibodies (Invitrogen, USA) were applied at a 1:200 dilution and incubated for 2 h at room temperature. The nuclei were stained with 4,6‐diamidino‐2‐phenylindole (DAPI, Yeasen, China). The sections were visualized and photographed under a Leica DMi8 laser confocal microscope (Leica, Germany).

### 2.8 Real‐time PCR analysis

To determine the levels of gene expression, total RNA was extracted from collected samples surrounding the injury lesion using TRIzol reagent (Invitrogen, USA). Subsequently, complementary DNA (cDNA) was synthesized from 1 μg of total RNA using the RevertAid First Strand cDNA Synthesis kit (Thermo, USA) in a 20 μL reaction volume. Quantitative PCR analysis was performed on a QuantStudio™ Dx Real‐Time PCR Instrument using synthetic primers (Table 2) and PowerUp SYBR Green Master Mix (Thermo, USA). The PCR protocol included denaturation at 95°C for 2 min, followed by 40 cycles of the amplification reaction (95°C for 15 s, 60°C for 15 s, and 72°C for 1 min). Each sample was evaluated in triplicate and normalized to the internal standard GAPDH. Relative quantification of mRNA expression was determined using the $2^{-∆∆C_{t}}$ method.

**TABLE 2:** Primer sequence for real‐time PCR.

Genes	Primer sequence (5′–3′)	
Rat‐RACK1	F: GCGGTGCTTGGCTCCCTAAG	R: CGGAGTGGTGGCGATCTGTG
Rat‐LC3	F: AACCAGGACAAGCAGGCAGATG	R: AGGCTTTCGTCTCTTCCACCATC
Rat‐Beclin1	F: ACTCTGGAGGTCTCGCTCTGG	R: GGTGCTGCTGGACGCCTTAG
Rat‐Atg5	F: TCAGGACGCCGAAGCATGACAC	R: TGGAATCTTCTGCCGCCTTGG
Rat‐GAPDH	F: CAGCAAGGATACTGAGAGCAAGAG	R: GGATGGAATTGTGAGGGAGATG

### 2.9 TUNEL staining

To quantify neuronal apoptosis in the pericontusional cortex 48 h after CCI, double staining with TUNEL (green) and for the neuron marker NeuN (red) was conducted. Briefly, frozen sections were first immunostained with mouse anti‐NeuN antibodies (1:200; EMD Millipore, MAB377, USA) at 4°C overnight and then subjected to TUNEL staining using a One Step TUNEL Apoptosis Assay kit (Beyotime, China) following the manufacturer's instructions. The number of TUNEL‐positive neurons in the pericontusional area of five sections per brain was observed under a confocal fluorescence microscope. The data are presented as the ratio of TUNEL‐positive neurons (%).

### 2.10 Nissl staining

Nissl staining was performed as previously described.^24^ Paraffin slices were successively dehydrated in 95% and 70% ethanol for 1 min, rinsed in distilled water for 30 s, and incubated with Nissl Staining Solution (Beyotime, China) for 10 min. Before being sealed with neutral balsam, the slices were soaked in 100% xylene for 2 min to achieve transparency. Finally, the slices were observed under a microscope, and images were collected. Damaged neurons were atrophic or contained vacuoles, whereas normal neurons had a relatively large and full soma, with large and round nuclei. The numbers of surviving neurons in three different fields in the pericontusional cortex were counted in five nonadjacent sections per brain, and the data are expressed as cells/field (400×).

### 2.11 Neurobehavioral assessment

#### 2.11.1 Modified neurological severity score (mNSS)

The mNSS was evaluated before CCI and at days 1, 3, 7, and 14 after CCI, as previously described.^25^ The mNSS is determined with motor and sensory functions, balance, and reflex tests. Neurological function was scored from 0 to 18 points. A higher score indicates more severe neurological impairment.

#### 2.11.2 Foot fault test

To evaluate sensorimotor function, the foot fault test was carried out before CCI and at 1, 3, 7, and 14 days after CCI. Rats were allowed to walk freely on a metal grid surface for 1 min, and a foot fault was recorded when a paw fell or slipped between the wires. The total number of steps taken and the total number of foot faults of each forelimb were counted. The data are expressed as the percentage of forelimb faults.

#### 2.11.3 Rotarod test

The rotarod test was used to evaluate motor function. Rats were placed on a rod that rotated with speeds starting at 4 rpm and accelerating to 40 rpm within 300 s. The test ended when the rats fell from the rod, and the latency was recorded. The data are expressed as the mean value from three trials. The test was performed before CCI and 1, 3, 7, and 14 days following CCI surgery.

#### 2.11.4 Morris water maze test

The Morris water maze test was used to evaluate spatial learning and memory 16–20 days post‐CCI. The experiment was conducted in a round pool with an escape platform located 2 cm under the water. Individual rats were subjected to four trials a day, in which they started in different quadrants with an interval of 60 s for spatial acquisition. If the rats did not find the platform within 60 s, they were guided to the platform, and the next quadrant test was carried out directly. The period and path length needed for each rat to find the hidden platform were observed. On day 21, the platform was removed for the probe trial. Escape latency, time spent in the target quadrant, number of platform crossings, and swimming speed within 60 s were recorded.

### 2.12 Contusion volume measurement

Cortical lesion volume was measured at 21 days after CCI. Coronal paraffin sections (5‐μm thick) were stained with hematoxylin and eosin reagent (Beyotime, China), and images were captured using a light microscope. Cortical lesion size was quantitatively analyzed with ImageJ software by outlining the injured brain area. The lesion volume was calculated based on the Cavalieri method of stereology^26^ and is presented as a volume percentage relative to the contralateral hemisphere.

### 2.13 Coimmunoprecipitation (Co‐IP)

Co‐IP was performed to examine protein interactions using SureBeads Protein A/G Magnetic Beads (MedChemExpress, USA). In brief, extracted proteins were incubated with mouse anti‐RACK1 antibodies (Santa, sc‐17754, USA) or normal rabbit IgG (negative control, Proteintech, 3000‐0‐AP, USA) overnight at 4°C. Then, the immune complexes were pulled down by incubation with protein A/G magnetic beads at 4°C for 4 h. Finally, microbeads were collected and washed, and immunoblotting was performed with rabbit anti‐LC3 (Proteintech, 14600‐1‐AP, China) and rabbit anti‐PERK (Cell Signaling, 3192S, USA) antibodies.

### 2.14 Statistical analysis

All the statistical analyses were performed using GraphPad Prism software (version 8.1.0, CA, USA). The results are expressed as the mean ± standard deviation (SD). All data used for analysis were tested for normality using Shapiro–Wilk test (Table S1). Comparisons between two groups were performed using Student's *t* test, and multiple comparisons were performed using a one‐way or two‐way ANOVA followed by post hoc Tukey's test. The correlations between the RACK1 and LC3‐II expression levels were assessed using Pearson correlation analysis. A value of *p* < 0.05 was considered to indicate a statistically significant difference.

## 3 RESULTS

### 3.1 RACK1 upregulation is correlated with autophagy induction after TBI

**FIGURE 1:**
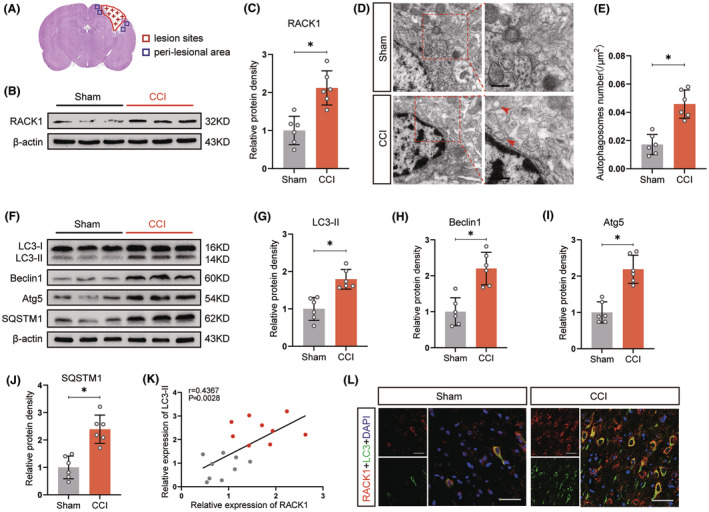
RACK1 upregulation is correlated with autophagy induction after TBI. (A) Schematic diagram showing the regions of interest that were selected for tissue collection and immunohistochemical image acquisition. Red areas represent lesion sites, and blue areas represent sample locations. (B, C) Representative immunoblots and quantification of RACK1 expression in the pericontusional cortex after TBI. β‐Actin was utilized as a loading control. (D) Electron microscopy images showing the autophagosomes (red arrows) in cortical neurons. Scale bars = 0.5 μm. (E) Quantitative analysis of the number of autophagosomes in the sham and CCI groups. (F–J) Representative immunoblots and quantification of LC3, Beclin‐1, Atg5, and SQSTM1 expression in the pericontusional cortex after TBI. (K) Pearson's correlation analysis between RACK1 and LC3‐II protein levels. (L) Representative immunofluorescence images of the colocalization of RACK1 (red) and LC3 (green) in the sham and CCI groups. Scale bars = 50 μm. The data are presented as the means ± SDs (*n* = 6 rats per group); *p < 0.05 indicates significant differences. Significance was calculated using Student's *t* test.

To evaluate the connection between RACK1 and autophagy induction after TBI, Western blotting analysis of RACK1 expression in the pericontusional cortex was performed. As shown in Figure 1A–C, the RACK1 protein level was markedly higher in the TBI group than in the control group (*p* < 0.05). Next, we investigated whether autophagy was initiated in brain regions with high RACK1 expression after TBI. TEM revealed that cortical neurons in TBI brains displayed increased numbers of autophagosomes with formed double‐membrane structures and enveloped cytoplasmic contents (Figure 1D,E, *p* < 0.05). Consistently, Western blotting analysis showed higher levels of critical autophagy‐related proteins, including LC3‐II, Beclin1, Atg5, and SQSTM1, in the TBI group than in the sham‐operated group (Figure 1F–J). Importantly, there was a positive correlation between RACK1 and LC3‐II protein expression levels in the pericontusional cortex (Figure 1K, *p* = 0.0028, *r* = 0.4367). In addition, immunofluorescence analysis revealed that both proteins were colocalized in the cytoplasm (Figure 1L). Altogether, these results suggested a correlation between RACK1 upregulation and autophagy induction after TBI.

### 3.2 Increased expression of RACK1 promotes neuronal autophagy after TBI

**FIGURE 2:**
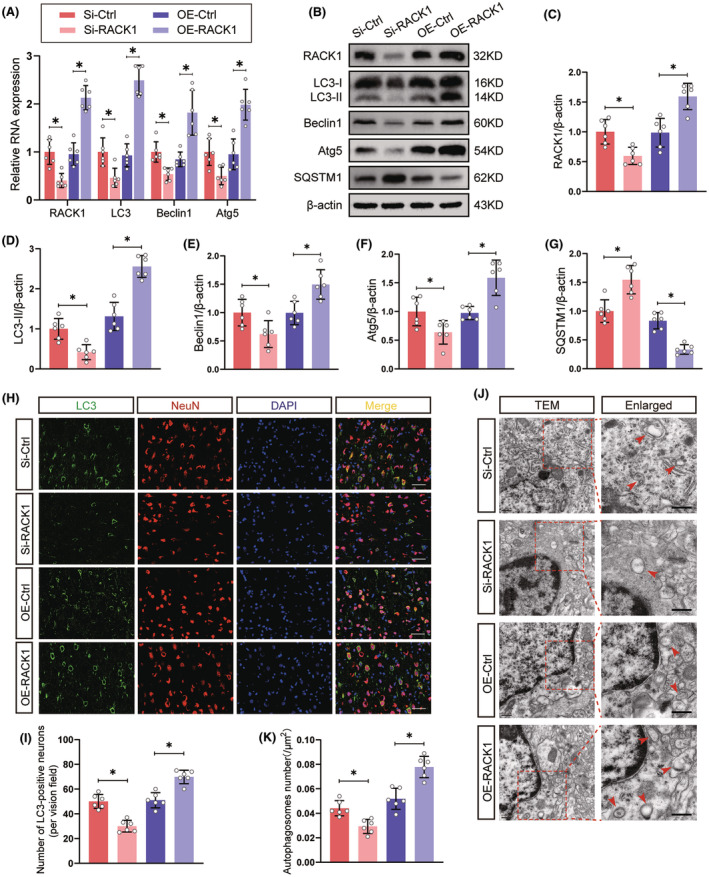
RACK1 promotes neuronal autophagy after TBI. Rats were intracerebroventricularly injected with RACK1 pcDNA or siRNA, followed by TBI model establishment. (A) Relative mRNA levels of RACK1 and autophagy‐related genes in the pericontusional cortex after TBI. The data were normalized to GAPDH expression. (B–G) Representative immunoblots and quantification of RACK1 and autophagy‐related protein expression in the pericontusional cortex following TBI. β‐Actin was utilized as a loading control. (H) Representative immunofluorescence images of the colocalization of LC3 (green)‐ and NeuN (red)‐labeled neurons in the pericontusional region. The nuclei were stained with DAPI (blue). Scale bars = 50 μm. (I) Quantitative analysis of the number of LC3‐positive neurons in each group. (J) Electron microscopy images showing the autophagosomes (red arrows) in cortical neurons. Scale bars = 0.5 μm. (K) Quantitative analysis of the number of autophagosomes in each group. The data are presented as the means ± SDs (*n* = 6 rats per group); *p < 0.05 indicates significant differences. Significance was calculated using one‐way ANOVA followed by Tukey's multiple comparison test.

To determine whether RACK1 upregulation might be involved in the induction of neuronal autophagy, we conducted gain‐ and loss‐of‐function experiments after the intracerebroventricular injection of RACK1 pcDNA (OE‐RACK1) or siRNA (si‐RACK1). As shown in Figure 2A–C, si‐RACK1 significantly decreased, whereas OE‐RACK1 significantly increased, RACK1 expression levels (*p* < 0.05). Moreover, we found that si‐RACK1 reversed TBI‐induced autophagy activation, as shown by the decrease in the expression of LC3‐II, Beclin1, and Atg5 and an increase in SQSTM1 protein levels. In contrast, overexpression of RACK1 exerted the opposite effect (Figure 2A–G). Next, the fluorescence colocalization of LC3 and the neuronal marker NeuN was determined to assess neuronal autophagy. We discovered that RACK1 knockdown by siRNA attenuated the punctate LC3 fluorescence signals in neurons, whereas overexpression of RACK1 further increased the punctate LC3 fluorescence signals in neurons post‐TBI (Figure 2H,I). Finally, the impact of RACK1 on the production of autophagosomes in cortical neurons was examined by electron microscopy. After si‐RACK1 treatment, the presence of autophagosomes decreased, whereas pretreatment with OE‐RACK1 resulted in a significant increase in autophagosome numbers (Figure 2J,K). Collectively, these findings indicated that increased RACK1 expression contributes to neuronal autophagy after TBI.

### 3.3 Inhibition of autophagy abolishes the RACK1‐mediated amelioration of neuronal apoptosis after TBI

**FIGURE 3:**
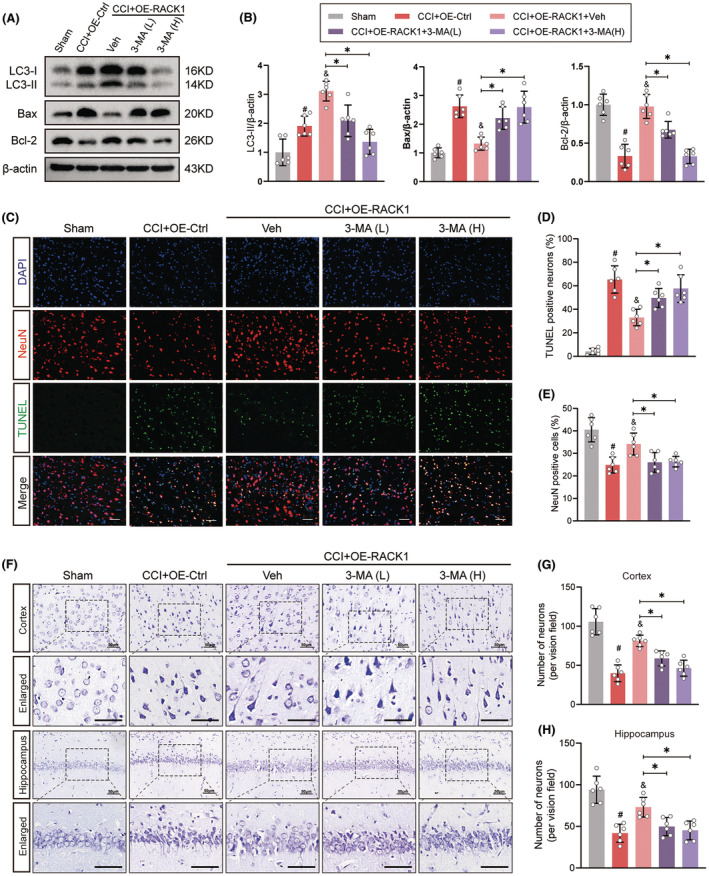
The autophagy inhibitor 3‐methyladenine abolishes RACK1‐mediated amelioration of neuronal apoptosis after TBI. Two doses of 3‐methyladenine (3‐MA, 400 and 600 nmol) were used to inhibit autophagy in the brains of TBI model rats. (A, B) Representative immunoblots and quantification of LC3, Bax, and Bcl‐2 expression in the pericontusional cortex 48 h following TBI. β‐Actin was utilized as a loading control. (C) Neuronal apoptosis in the pericontusional region was determined using TUNEL (green)‐NeuN (red) double staining. Scale bars = 50 μm. (D, E) Quantitative analysis of the proportion of TUNEL‐positive neurons and NeuN‐positive cells in each group. (F) Representative photomicrographs of Nissl staining showing surviving neurons in the pericontusional region and hippocampus CA1 region. Scale bars = 50 μm. (G, H) Quantitative analysis of the number of surviving neurons in the pericontusional cortex and hippocampus CA1 region in each group. The data are presented as the means ± SDs (*n* = 6 rats per group); *p < 0.05 versus the indicated group; ^#^ *p* < 0.05 versus sham; ^&^ *p* < 0.05 versus CCI + OE‐Ctrl. Significance was calculated using one‐way ANOVA followed by Tukey's multiple comparison test.

**FIGURE 4:**
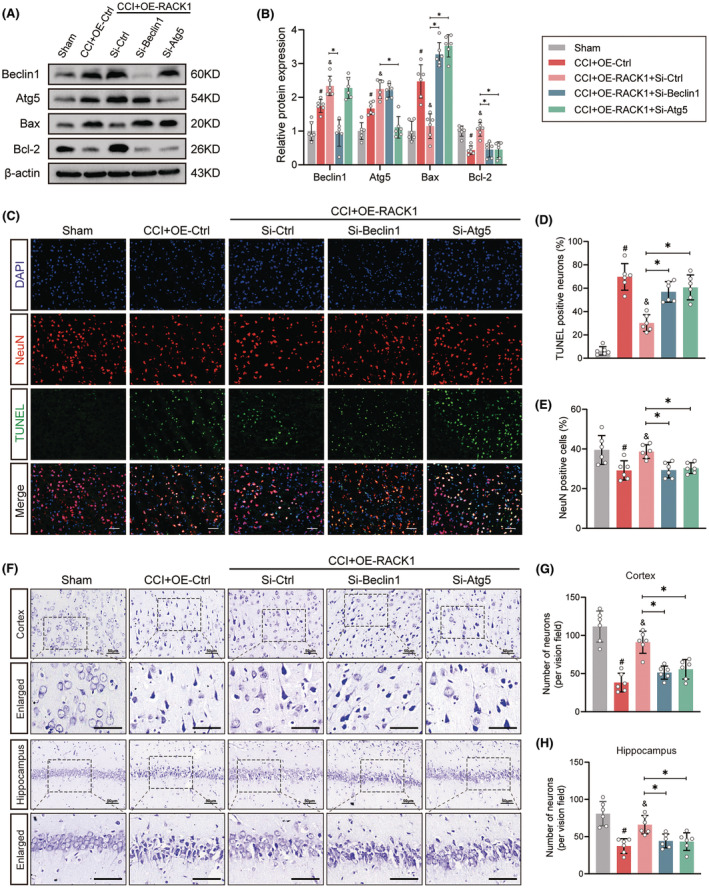
Knockdown of Beclin1 or Atg5 reverses the RACK1‐mediated amelioration of neuronal apoptosis after TBI. (A, B) Representative immunoblots and quantification of Beclin1, Atg5, Bax, and Bcl‐2 expression in the pericontusional cortex at 48 h following TBI. β‐Actin was utilized as a loading control. (C) Representative images of the colocalization of TUNEL (green) with neurons (NeuN, red) in the pericontusional region. Scale bars = 50 μm. (D, E) Quantitative analysis of the proportion of TUNEL‐positive neurons and NeuN‐positive cells in each group. (F) Representative photomicrographs of Nissl staining showing surviving neurons in the pericontusional region and hippocampus CA1 region. Scale bars = 50 μm. (G, H) Quantitative analysis of the number of surviving neurons in the pericontusional cortex and hippocampus CA1 region in each group. The data are presented as the means ± SDs (*n* = 6 rats per group); *p < 0.05 versus the indicated group; ^#^ *p* < 0.05 versus sham; ^&^ *p* < 0.05 versus CCI + OE‐Ctrl. Significance was calculated using one‐way ANOVA followed by Tukey's multiple comparison test.

Our previous research showed that RACK1 upregulation plays a critical role in neuronal survival after TBI.^16^ To determine the contribution of the autophagic mechanism to RACK1‐mediated neuroprotection, we utilized the known autophagy inhibitor 3‐MA, which blocks the formation of autophagosomes at the initiation stage. The effect of 3‐MA in inhibiting autophagy was confirmed by decreased LC3‐II expression in brain tissues (Figure 3A,B). Moreover, Western blotting results showed that OE‐RACK1 decreased the expression of Bax and elevated the expression of Bcl‐2 after TBI, while cotreatment with 3‐MA markedly reversed these effects (Figure 3A,B, *p* < 0.05). Next, apoptotic and surviving neurons were assessed by TUNEL and Nissl staining at 48 h after TBI. As shown in Figure 3C–H, TBI caused a significant increase in the proportion of TUNEL‐positive neurons in the pericontusional cortex and a significant decrease in the number of surviving neurons in the pericontusional cortex and hippocampus CA1 region. These changes were ameliorated by OE‐RACK1, but the beneficial effect of OE‐RACK1 was abolished by cotreatment with 3‐MA (*p* < 0.05). Moreover, we used siRNA targeting Beclin1 or Atg5 to inhibit the induction of autophagy. Compared to OE‐RACK1 treatment alone, both siRNAs significantly reversed the antiapoptotic effects of OE‐RACK1 on neurons at 48 h after TBI (Figure 4A–H, *p* < 0.05). Together, these results revealed that RACK1 ameliorates TBI‐induced neuronal apoptosis by enhancing autophagy.

### 3.4 Inhibition of autophagy reverses the protective effects of RACK1 on neurological deficits and tissue loss after TBI

**FIGURE 5:**
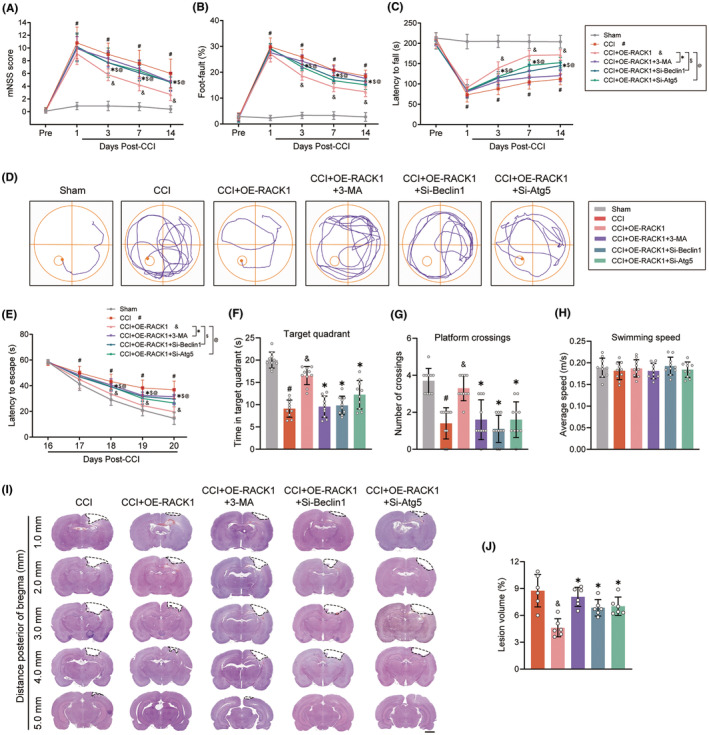
Autophagy inhibition reverses the protective effects of RACK1 on neurological deficits and tissue loss after TBI. Modified neurological severity score (mNSS) (A), foot‐fault test (B), and rotarod test (C) were conducted at different time points to evaluate the motor function of rats in each group. (D) Representative swimming tracks during the probe trial of the Morris water maze. (E) Escape latencies on days 16 to 20 after TBI are shown in the line graph. Quantification of time spent in the target quadrant (F), the number of platform crossings (G), and swimming speed (H) on day 21 after TBI. (I) Representative images of H&E‐stained brain sections on day 21 after TBI. The dotted black line indicates the lesion area. Scar bar = 2 mm. (J) Lesion volume calculated as a percent of the contralateral hemisphere. The data are presented as the means ± SDs (*n* = 8–10 rats per group); *p < 0.05 versus CCI + OE‐RACK1; ^$^ *p* < 0.05 versus CCI + OE‐RACK1; ^@^ *p* < 0.05 versus CCI + OE‐RACK1; ^#^ *p* < 0.05 versus sham; ^&^ *p* < 0.05 versus CCI. Significance was calculated using two‐way ANOVA followed by Tukey's multiple comparison test.

To further elucidate whether RACK1 upregulation exerts its neuroprotective effects against TBI by regulating autophagy, we assessed neurological function. As presented in Figure 5A–C, rats suffering from TBI exhibited deficits in motor function, as shown by elevated mNSS scores, a greater percentage of foot faults, and a shorter latency to fall from the rotating rod on days 1, 3, 7, and 14 postsurgery. Administration of OE‐RACK1 significantly improved motor function on days 3, 7, and 14 postsurgery compared with the TBI group, and this effect was obviously reversed by cotreatment with 3‐MA or siRNAs targeting Beclin1 and Atg5 (all *p* < 0.05). Next, the Morris water maze test was used to evaluate spatial cognitive functions 16–21 days postsurgery. As shown in Figure 5D–G, TBI model rats treated with OE‐RACK1 had significantly improved spatial learning and memory, which resulted in decreased escape latency, longer time spent in the target quadrant, and more crossings of the platform area compared to the TBI group. However, the beneficial impact of OE‐RACK1 was abolished by treatment with 3‐MA or siRNAs targeting Beclin1 and Atg5 (all *p* < 0.05). The swimming speed was not significantly different among the groups (Figure 5H, *p* > 0.05). In addition, H&E staining showed that OE‐RACK1 treatment significantly reduced TBI‐induced cerebral lesion volumes, whereas this effect was reversed by inhibiting autophagy with 3‐MA or siRNAs targeting Beclin1 and Atg5 (Figure 5I,J, *p* < 0.05). These data suggested that autophagy induction is involved in the neuroprotective effects of RACK1 against behavioral deficits and tissue loss after TBI.

### 3.5 RACK1 interacts with PERK and activates PERK signaling after TBI

**FIGURE 6:**
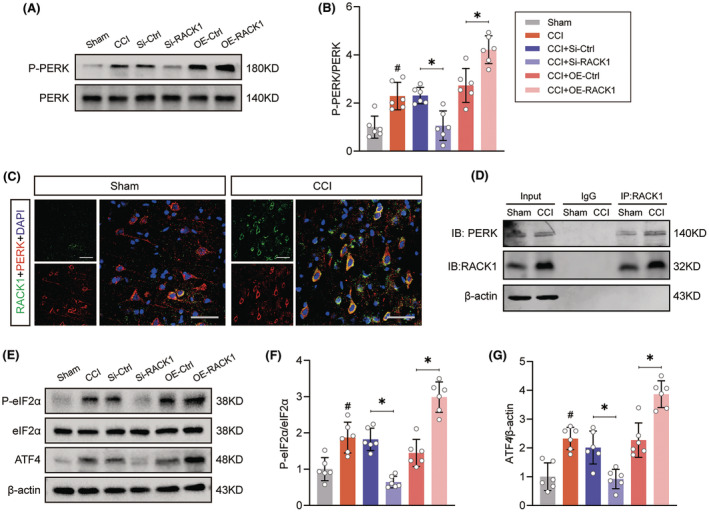
RACK1 interacts with PERK and activates PERK signaling after TBI. (A, B) Representative immunoblots and quantification of phosphorylated PERK levels in the pericontusional cortex after TBI. (C) Representative immunofluorescence images of the colocalization of RACK1 (green) and PERK (red) in the sham and CCI groups. Scale bars = 50 μm. (D) The RACK1 and PERK interaction was analyzed using coimmunoprecipitation (Co‐IP) with an anti‐RACK1 antibody or control IgG. (E–G) Representative immunoblots and quantification of phosphorylated eIF2α and ATF4 levels in the pericontusional cortex after TBI. β‐Actin was utilized as a loading control. The data are presented as the means ± SDs (*n* = 6 rats per group); *p < 0.05 versus the indicated group; ^#^ *p* < 0.05 versus sham. Significance was calculated using one‐way ANOVA followed by Tukey's multiple comparison test.

Based on accumulating evidence, RACK1 is implicated in the activation of the unfolded protein response (UPR).^16^, ^27^ As a primary branch of the UPR, PERK signaling has been shown to be a key regulator of autophagy.^28^ To elucidate the possible molecular mechanisms underlying RACK1‐modulated autophagy, the effect of RACK1 on PERK signaling was assessed. Western blotting analysis demonstrated that the levels of phosphorylated PERK significantly increased at 48 h after TBI compared to those in the sham group (Figure 6A,B, *p* < 0.05). Knockdown of RACK1 suppressed PERK phosphorylation, whereas RACK1 overexpression further enhanced PERK phosphorylation after TBI. Next, to validate the protein interaction between RACK1 and PERK, double‐staining and Co‐IP analyses were carried out. As expected, PERK colocalized with elevated RACK1 protein levels in the cytoplasm postinjury (Figure 6C). Moreover, in a reciprocal Co‐IP with RACK1, PERK was detected in the immunoprecipitated complex (Figure 6D). Then, we investigated signaling downstream of the PERK pathway by western blotting. As shown in Figure 6E–G, RACK1 knockdown reversed the TBI‐induced increase in the phosphorylated eIF2α and ATF4 protein levels. In contrast, RACK1 overexpression exerted the opposite effect. Together, these observations suggest that RACK1 interacts with PERK and promotes PERK signaling activation following TBI.

### 3.6 RACK1 promotes neuronal autophagy by activating PERK signaling after TBI

**FIGURE 7:**
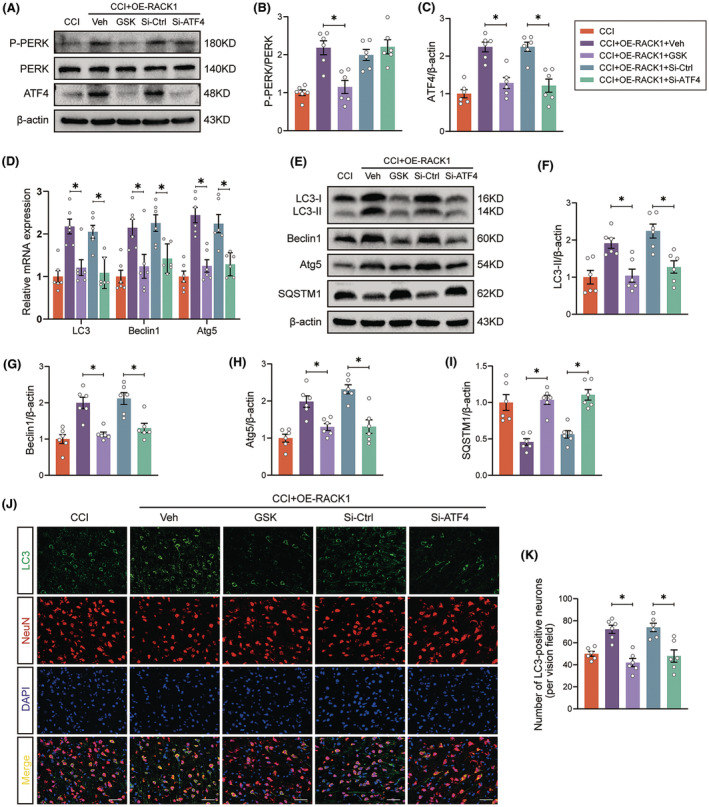
RACK1 Promotes neuronal autophagy by activating PERK signaling after TBI. The PERK inhibitor GSK2606414 (GSK, 90 μg/μL) and siRNA targeting ATF4 (si‐ATF4) were used to block the PERK signaling pathway. (A–C) Representative immunoblots and quantification of phosphorylated PERK and ATF4 expression in the pericontusional cortex after TBI. β‐Actin was utilized as a loading control. (D) Relative mRNA levels of autophagy‐related genes in the pericontusional cortex after TBI. The data were normalized to GAPDH expression. (E–I) Representative immunoblots and quantification of RACK1 and autophagy‐related protein expression in the pericontusional cortex after TBI. β‐Actin was utilized as a loading control. (J) Representative immunofluorescence images of the colocalization of LC3 (green)‐ and NeuN (red)‐labeled neurons in the pericontusional region in the indicated groups. Scale bars = 50 μm. (K) Quantitative analysis of the number of LC3‐positive neurons in each group. The data are presented as the means ± SDs (*n* = 6 rats per group); *p < 0.05 versus the indicated group. Significance was calculated using one‐way ANOVA followed by Tukey's multiple comparison test.

To further verify whether PERK signaling is needed for RACK1‐mediated neuronal autophagy in TBI, we applied the specific PERK inhibitor GSK2606414 and siRNA targeting ATF4 (si‐ATF4) to block the PERK/eIF2α/ATF4 pathway. As shown in Figure 7A–C, GSK2606414 efficiently inhibited the levels of phosphorylated PERK and downstream ATF4 expression (all *p* < 0.05). More importantly, the RACK1 overexpression‐mediated increase in the mRNA and protein levels of LC3‐II, Beclin1, and Atg5 and the decrease in SQSTM1 expression were reversed by GSK2606414 treatment (Figure 7D–I, all *p* < 0.05). Similar results were observed after knockdown of ATF4 with si‐ATF4 (Figure 7A–I). In addition, consistent with the western blot findings, fluorescence staining revealed that both GSK2606414 treatment and ATF4 knockdown efficiently abolished the OE‐RACK1‐induced increase in the punctate LC3 fluorescence signals in neurons post‐TBI (Figure 7J,K). Altogether, these results indicated that RACK1 promotes neuronal autophagy, at least partially, by activating PERK signaling.

## 4 DISCUSSION

TBI is a devastating clinical event for which there are no effective treatments. Among the various pathological processes that contribute to secondary injury, neuronal apoptosis has been reported to be an essential process that might explain the severe impact of TBI on short‐term and long‐term outcomes.^3^, ^29^ Therefore, preventing irreversible damage by targeting neuronal apoptosis pathways may be a promising strategy for treating TBI. The present study evaluated the regulatory effect of RACK1 on autophagy and its significance in TBI‐induced neuronal injury. Our data demonstrated that endogenous RACK1 was upregulated and associated with autophagy induction after TBI. Knockdown of RACK1 impaired, whereas RACK1 overexpression in TBI model rats enhanced, neuronal autophagy. In addition, inhibition of autophagy reversed the protective effects of RACK1 on neuronal apoptosis, neurological deficits, and cortical tissue loss caused by TBI. In the mechanistic analysis, we observed that modulation of the PERK signaling pathway is involved in the RACK1‐mediated induction of autophagy. Our current data suggest that RACK1 promotes autophagy induction partly through the PERK signaling pathway, and this mechanism contributes to the protective effects of RACK1 against neuronal damage after TBI.

Autophagy is an evolutionarily conserved catabolic event that maintains cell homeostasis and is induced in response to various stimuli.^30^ Several findings have indicated that RACK1 is involved in the regulation of autophagy.^17^, ^31^, ^32^ In a previous study, it was shown that endogenous RACK1 partially colocalized with early autophagic structures, and deletion of RACK1 decreased starvation‐induced autophagy in Drosophila, suggesting a role of RACK1 in autophagy in response to starvation.^31^ In addition, RACK1 has been shown to act as a key element that enhances the assembly of the Atg14L‐Beclin 1‐Vps34‐Vps15 complex, which promotes autophagy initiation and autophagosome formation in hepatocytes.^32^ More recently, RACK1 was demonstrated to be involved in the proliferation and survival of colon cancer cells by facilitating autophagy initiation.^17^ Consistent with these results, in the current study, we found that increased autophagy was correlated with the upregulation of RACK1. More importantly, RACK1 knockdown significantly inhibited TBI‐induced increases in the levels of autophagy‐related proteins, such as LC3‐II and Atg5, and the formation of autophagosomes in neurons, while overexpression of RACK1 exerted the opposite effects, suggesting that RACK1 might be an essential positive regulator of neuronal autophagy after TBI.

Autophagic dysfunction can occur in various phases of the autophagic process, including autophagosome formation, autophagosome‐lysosome fusion, and lysosomal degradation.^33^ The SQSTM1 protein serves as a bridge molecule that connects LC3‐decorated autophagosomes to ubiquitin‐bound protein aggregates, and it is ultimately degraded inside autolysosomes. Impaired autophagic activity results in an increase in SQSTM1; therefore, SQSTM1 levels have been utilized to monitor the autophagic flux.^34^ The observations of this study are consistent with those of previous reports showing concomitant accumulation of both LC3‐II and SQSTM1 after TBI.^35^, ^36^ Additionally, it is noteworthy that RACK1 knockdown exacerbated, whereas RACK1 overexpression reduced, SQSTM1 aggregation in the injured cortex of TBI model rats, suggesting that RACK1 might be involved in the regulation of the autophagic flux. However, the use of immunoblotting to evaluate the effect of RACK1 on the autophagic flux provides only one‐way evidence, and direct evidence of changes in the autophagic flux is lacking. Therefore, additional methods, such as electron microscopic analysis or tandem fluorescent‐tagged LC3 (mRFP‐GFP‐LC3), are required to visualize autophagosome formation in vivo.

In recent years, there has been a great deal of interest in the crosstalk between autophagy and apoptosis, as both processes are generally induced by stress.^37^ Whether autophagy induction promotes cell survival after brain trauma or, on the contrary, aggravates cell death is still controversial. Autophagy was initially considered to be an upstream mechanism that induces cell death because Beclin1/TUNEL‐double‐positive cells were observed at the lesion site.^38^ This notion was then supported by several studies that demonstrated that the inhibition of autophagy by neuroprotective drugs could reduce neural tissue damage in a TBI model.^39^, ^40^ More recently, however, accumulating evidence suggests that TBI‐induced enhancement of autophagy could serve as a prosurvival mechanism by eliminating damaged intracellular substances. Beclin1‐knockout mice with acute brain damage exhibited defective autophagy and increased neuronal degeneration in the peri‐injured area.^41^ Moreover, several studies have reported that autophagy‐enhancing drugs can reduce apoptosis, while autophagy inhibitors exacerbate apoptosis after TBI.^42^ Given that both increased RACK1 and enhanced autophagy are reportedly involved in neuroprotection after TBI,^16^, ^42^ we hypothesized that there was likely a close correlation between these phenomena in the facilitation of post‐TBI recovery. The present study showed that inhibition of autophagy by 3‐MA effectively suppressed RACK1‐mediated neuroprotective effects, as shown by improved neurological outcomes, reduced neuronal apoptosis, and decreased cortical tissue loss; these results were consistent with previous studies that support the protective role of autophagy in TBI rather than a cause of neuronal apoptosis after brain trauma. Beclin1 and Atg5 are two critical proteins that are involved in autophagosome formation.^43^ Therefore, we genetically inhibited both proteins to further study the involvement of autophagy in RACK1‐regulated neuronal survival. We confirmed that knocking down Beclin1 or Atg5 to inhibit autophagy partially blocked the RACK1 overexpression‐mediated amelioration of neural damage and functional outcomes. These results strongly suggest that the induction of Beclin1‐ and Atg5‐mediated autophagy is associated with the neuroprotective effects of RACK1 in TBI.

By the combined use of coimmunoprecipitation and immunofluorescence technology, we show for the first time that RACK1 interacts with the ER kinase PERK. As one of three arms of the UPR response, PERK's main function is to inhibit the initiation of translation by increasing the phosphorylation of the eIF2α initiation factor subunit. This in turn triggers a selective translation and transcription program that enables cells to cope with ER stress.^44^ Notably, recent investigations have revealed that PERK signaling also functions as a stress response that induces autophagy as a survival mechanism in response to multiple cellular stimuli, such as hypoxia, nutrient deprivation, or radiation.^28^ Our results were consistent with published work^45^ and demonstrated that PERK phosphorylation was significantly increased in the brains of TBI model rats. Furthermore, the results of our gain‐ and loss‐of‐function experiments indicate that RACK1 serves as an upstream regulator of PERK signaling, which can enhance the phosphorylation of eIF2α and consequently lead to increased expression of the transcription factor ATF4.ATF4, which is the effector of the PERK/eIF2α pathway, has been demonstrated to play an essential role in autophagy induction by regulating the transcription of multiple autophagy‐related genes, including LC3, Atg5, and Beclin1.^46^ These results prompted us to investigate whether the mechanism by which RACK1 triggers autophagy induction during TBI involves its regulation of PERK signaling. As expected, our results showed that both treatment with the PERK inhibitor GSK2606414 and knockdown of ATF4 effectively abolished the RACK1 overexpression‐mediated induction of autophagy, indicating that RACK1 promoted autophagy after TBI at least partially by activating PERK signaling. Indeed, PERK signaling is not the only pathway that is involved in RACK1‐induced autophagy. Previous studies showed that RACK1 may directly bind to Atg14L and Beclin1 upon its phosphorylation at the T50 site, thereby promoting the initiation of autophagosome formation.^13^, ^32^ In addition, RACK1 has been proven to interact with Atg5, which is essential for starvation‐induced autophagy.^47^ Therefore, whether RACK1 affects the progression of autophagy by directly targeting autophagy‐related proteins will be the focus of our next study.

**FIGURE 8:**
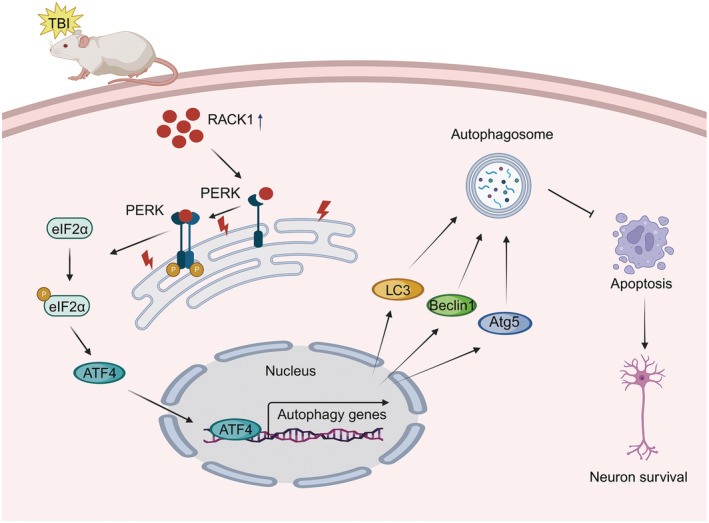
Schematic illustrating the proposed mechanism by which the PERK pathway modulates autophagy during RACK1‐mediated neuroprotection after TBI. TBI upregulates RACK1, which interacts with PERK and activates the PERK/eIF2α/ATF4 signaling pathway, thereby promoting autophagy induction by increasing the transcription of multiple autophagy‐related genes, including LC3, Atg5, and Beclin1. Thus, RACK1 upregulation alleviates neuronal apoptosis after TBI.

To our knowledge, this may be the first report on the regulatory effect of RACK1 on autophagy induction under conditions of brain trauma. Overall, the present study demonstrated that RACK1 could activate autophagy in the cerebral cortex and thus alleviate neuronal damage induced by TBI, and the underlying mechanism appears to partly involve activation of the PERK/eIF2α/ATF4 signaling pathway (Figure 8). Our findings provide new insights into the mechanisms underlying the modulatory effect of RACK1 on autophagy and may provide potential therapeutic targets for the treatment of TBI.

## AUTHOR CONTRIBUTIONS

**Haibo Ni:** Investigation, Data curation, Funding acquisition, Investigation, Writing – original draft. **Qin Rui:** Methodology, Investigation, Software, Funding acquisition, Writing – original draft. **Xugang Kan:** Investigation, Software, Validation, Visualization. **Yang Zhang:** Validation, Visualization. **Weiwei Zhai:** Conceptualization, Formal analysis, Investigation. **Baole Zhang:** Validation, Funding acquisition, Project administration, Writing – review & editing. **Zhengquan Yu:** Conceptualization, Formal analysis, Funding acquisition, Project administration, Resources, Supervision, Validation, Writing – review & editing.

## FUNDING INFORMATION

This research was funded by the National Natural Science Foundation of China (No. 81901254 and 82201538), the Project of Suzhou Health Talent Training (No. GSWS2020105), Six Talent Peaks in Jiangsu Province (SWYY‐088, 2018 to B.L. Zhang), the 333 Project of Jiangsu Province (2022 to B.L. Zhang), and Qing Lan Project in Jiangsu Province (2017 to B.L. Zhang).

## CONFLICT OF INTEREST STATEMENT

The authors declare no conflicts of interest.

## Supporting information


Table S1

## Data Availability

The data that support the findings of this study are available in the paper or from the corresponding author upon reasonable request.
