# Supplementary material for: RACK1 promotes autophagy via the PERK signaling pathway to protect against traumatic brain injury in rats
Source: CNS Neurosci Ther. 2024 Mar 26;30(3):e14691. doi: 10.1111/cns.14691 (PMC10966134; doi:10.1111/cns.14691)
Supplement: Supplementary file 1 — Table S1 [file CNS-30-e14691-s001.docx]

**Table S1. Statistical analysis**

| FIGURE | N | DATA STRUCTURE | TEST USED | STATISTIC | P VALUE |
| --- | --- | --- | --- | --- | --- |
| 1C | 6 | Normal  distribution | Unpaired t test, two-tailed | t = 4.683 | 0.0009 |
| 1E | 6 | Normal  distribution | Unpaired t test, two-tailed | t = 5.704 | 0.0002 |
| 1G | 6 | Normal  distribution | Unpaired t test, two-tailed | t = 4.801 | 0.0007 |
| 1H | 6 | Normal  distribution | Unpaired t test, two-tailed | t = 4.942 | 0.0006 |
| 1I | 6 | Normal  distribution | Unpaired t test, two-tailed | t = 6.007 | 0.0001 |
| 1J | 6 | Normal  distribution | Unpaired t test, two-tailed | t = 5.136 | 0.0004 |
| 1K | 18 | Normal  distribution | Simple linear regression | r = 0.4367 | 0.0028 |
| 2A. RACK1 | 6 | Normal  distribution | One-way ANOVA, Tukey post hoc | F (3, 20) = 60.03 | Si-Ctrl vs. Si-RACK1 = 0.0013;  OE-Ctrl vs. OE-RACK1 <0.0001; |
| 2A. LC3 | 6 | Normal  distribution | One-way ANOVA, Tukey post hoc | F (3, 20) = 66.06 | Si-Ctrl vs. Si-RACK1 = 0.0130;  OE-Ctrl vs. OE-RACK1 <0.0001; |
| 2A. Beclin1 | 6 | Normal  distribution | One-way ANOVA, Tukey post hoc | F (3, 20) = 23.62 | Si-Ctrl vs. Si-RACK1 = 0.0168;  OE-Ctrl vs. OE-RACK1 <0.0001; |
| 2A.Atg5 | 6 | Normal  distribution | One-way ANOVA, Tukey post hoc | F (3, 20) = 29.60 | Si-Ctrl vs. Si-RACK1 = 0.0082;  OE-Ctrl vs. OE-RACK1 <0.0001; |
| 2C | 6 | Normal  distribution | One-way ANOVA, Tukey post hoc | F (3, 20) = 60.54 | Si-Ctrl vs. Si-RACK1 = 0.0221;  OE-Ctrl vs. OE-RACK1 <0.0001; |
| 2D | 6 | Normal  distribution | One-way ANOVA, Tukey post hoc | F (3, 20) = 63.00 | Si-Ctrl vs. Si-RACK1 = 0.0088;  OE-Ctrl vs. OE-RACK1 = 0.0022; |
| 2E | 6 | Normal  distribution | One-way ANOVA, Tukey post hoc | F (3, 20) = 48.56 | Si-Ctrl vs. Si-RACK1 = 0.0086;  OE-Ctrl vs. OE-RACK1 = 0.0003; |
| 2F | 6 | Normal  distribution | One-way ANOVA, Tukey post hoc | F (3, 20) = 21.96 | Si-Ctrl vs. Si-RACK1 = 0.0302;  OE-Ctrl vs. OE-RACK1 = 0.0062; |
| 2G | 6 | Normal  distribution | One-way ANOVA, Tukey post hoc | F (3, 20) = 71.52 | Si-Ctrl vs. Si-RACK1 = 0.0084;  OE-Ctrl vs. OE-RACK1 = 0.0007; |
| 2I | 6 | Normal  distribution | One-way ANOVA, Tukey post hoc | F (3, 20) = 44.90 | Si-Ctrl vs. Si-RACK1 = 0.0088;  OE-Ctrl vs. OE-RACK1 = 0.0036; |
| 2K | 6 | Normal  distribution | One-way ANOVA, Tukey post hoc | F (3, 20) = 62.03 | Si-Ctrl vs. Si-RACK1 = 0.0276;  OE-Ctrl vs. OE-RACK1 = 0.0077; |
| 3B.LC3 | 6 | Normal  distribution | One-way ANOVA, Tukey post hoc | F (4, 25) = 21.22 | Sham vs. CCI+OE-Ctrl = 0.0094;  CCI+OE-Ctrl vs. CCI+OE-RACK1+Veh = 0.0005;  CCI+OE-RACK1+Veh vs. CCI+OE-RACK1+3-MA(L) = 0.0030;  CCI+OE-RACK1+Veh vs. CCI+OE-RACK1+3-MA(H) <0.0001; |
| 3B.bax | 6 | Normal  distribution | One-way ANOVA, Tukey post hoc | F (4, 25) = 23.89 | Sham vs. CCI+OE-Ctrl) <0.0001;  CCI+OE-Ctrl vs. CCI+OE-RACK1+Veh <0.0001;  CCI+OE-RACK1+Veh vs. CCI+OE-RACK1+3-MA(L) = 0.0031;  CCI+OE-RACK1+Veh vs. CCI+OE-RACK1+3-MA(H) <0.0001; |
| 3B.Bcl-2 | 6 | Normal  distribution | One-way ANOVA, Tukey post hoc | F (4, 25) = 37.33 | Sham vs. CCI+OE-Ctrl) <0.0001;  CCI+OE-Ctrl vs. CCI+OE-RACK1+Veh <0.0001;  CCI+OE-RACK1+Veh vs. CCI+OE-RACK1+3-MA(L) = 0.0043;  CCI+OE-RACK1+Veh vs. CCI+OE-RACK1+3-MA(H) <0.0001; |
| 3D | 6 | Normal  distribution | One-way ANOVA, Tukey post hoc | F (4, 25) = 44.88 | Sham vs. CCI+OE-Ctrl) <0.0001;  CCI+OE-Ctrl vs. CCI+OE-RACK1+Veh <0.0001;  CCI+OE-RACK1+Veh vs. CCI+OE-RACK1+3-MA(L) = 0.0249;  CCI+OE-RACK1+Veh vs. CCI+OE-RACK1+3-MA(H) = 0.0005; |
| 3E | 6 | Normal  distribution | One-way ANOVA, Tukey post hoc | F (4, 25) = 15.38 | Sham vs. CCI+OE-Ctrl) <0.0001;  CCI+OE-Ctrl vs. CCI+OE-RACK1+Veh = 0.0063;  CCI+OE-RACK1+Veh vs. CCI+OE-RACK1+3-MA(L) = 0.0199;  CCI+OE-RACK1+Veh vs. CCI+OE-RACK1+3-MA(H) = 0.0272; |
| 3G | 6 | Normal  distribution | One-way ANOVA, Tukey post hoc | F (4, 25) = 34.01 | Sham vs. CCI+OE-Ctrl) <0.0001;  CCI+OE-Ctrl vs. CCI+OE-RACK1+Veh<0.0001;  CCI+OE-RACK1+Veh vs. CCI+OE-RACK1+3-MA(L) = 0.0165;  CCI+OE-RACK1+Veh vs. CCI+OE-RACK1+3-MA(H) = 0.0001; |
| 3H | 6 | Normal  distribution | One-way ANOVA, Tukey post hoc | F (4, 25) = 19.27 | Sham vs. CCI+OE-Ctrl) <0.0001;  CCI+OE-Ctrl vs. CCI+OE-RACK1+Veh = 0.0017;  CCI+OE-RACK1+Veh vs. CCI+OE-RACK1+3-MA(L) = 0.0245;  CCI+OE-RACK1+Veh vs. CCI+OE-RACK1+3-MA(H) = 0.0055; |
| 4B.Beclin1 | 6 | Normal  distribution | One-way ANOVA, Tukey post hoc | F (4, 25) = 30.52 | Sham vs. CCI+OE-Ctrl = 0.0018;  CCI+OE-Ctrl vs. CCI+OE-RACK1+Si-Ctrl = 0.0151;  CCI+OE-RACK1+Si-Ctrl vs. CCI+OE-RACK1+Si-Beclin1 =0.0165; |
| 4B.Atg5 | 6 | Normal  distribution | One-way ANOVA, Tukey post hoc | F (4, 25) = 33.05 | Sham vs. CCI+OE-Ctrl) = 0.0008;  CCI+OE-Ctrl vs. CCI+OE-RACK1+Si-Ctrl = 0.0043;  CCI+OE-RACK1+Si-Ctrl vs. CCI+OE-RACK1+Si-Atg5 <0.0001; |
| 4B.Bax | 6 | Normal  distribution | One-way ANOVA, Tukey post hoc | F (4, 25) = 59.62 | Sham vs. CCI+OE-Ctrl <0.0001;  CCI+OE-Ctrl vs. CCI+OE-RACK1+Si-Ctrl <0.0001;  CCI+OE-RACK1+Si-Ctrl vs. CCI+OE-RACK1+Si-Beclin1 <0.0001;  CCI+OE-RACK1+Si-Ctrl vs. CCI+OE-RACK1+Si-Atg5 <0.0001; |
| 4B.Bcl-2 | 6 | Normal  distribution | One-way ANOVA, Tukey post hoc | F (4, 25) = 16.66 | Sham vs. CCI+OE-Ctrl) = 0.0002;  CCI+OE-Ctrl vs. CCI+OE-RACK1+Si-Ctrl <0.0001;  CCI+OE-RACK1+Si-Ctrl vs. CCI+OE-RACK1+Si-Beclin1 =0.0001;  CCI+OE-RACK1+Si-Ctrl vs. CCI+OE-RACK1+Si-Atg5 =0.0001; |
| 4D | 6 | Normal  distribution | One-way ANOVA, Tukey post hoc | F (4, 25) = 53.25 | Sham vs. CCI+OE-Ctrl) <0.0001;  CCI+OE-Ctrl vs. CCI+OE-RACK1+Si-Ctrl <0.0001;  CCI+OE-RACK1+Si-Ctrl vs. CCI+OE-RACK1+Si-Beclin1 =0.0002;  CCI+OE-RACK1+Si-Ctrl vs. CCI+OE-RACK1+Si-Atg5 <0.0001; |
| 4E | 6 | Normal  distribution | One-way ANOVA, Tukey post hoc | F (4, 25) = 7.334 | Sham vs. CCI+OE-Ctrl) = 0.0068;  CCI+OE-Ctrl vs. CCI+OE-RACK1+Si-Ctrl = 0.0143;  CCI+OE-RACK1+Si-Ctrl vs. CCI+OE-RACK1+Si-Beclin1 =0.0165;  CCI+OE-RACK1+Si-Ctrl vs. CCI+OE-RACK1+Si-Atg5 =0.0382; |
| 4G | 6 | Normal  distribution | One-way ANOVA, Tukey post hoc | F (4, 25) = 27.72 | Sham vs. CCI+OE-Ctrl) <0.0001;  CCI+OE-Ctrl vs. CCI+OE-RACK1+Si-Ctrl <0.0001;  CCI+OE-RACK1+Si-Ctrl vs. CCI+OE-RACK1+Si-Beclin1 =0.0005;  CCI+OE-RACK1+Si-Ctrl vs. CCI+OE-RACK1+Si-Atg5 =0.0018; |
| 4H | 6 | Normal  distribution | One-way ANOVA, Tukey post hoc | F (4, 25) = 13.65 | Sham vs. CCI+OE-Ctrl) <0.0001;  CCI+OE-Ctrl vs. CCI+OE-RACK1+Si-Ctrl =0.0034;  CCI+OE-RACK1+Si-Ctrl vs. CCI+OE-RACK1+Si-Beclin1 =0.0347;  CCI+OE-RACK1+Si-Ctrl vs. CCI+OE-RACK1+Si-Atg5 =0.0266; |
| 5A | 10 | Normal  distribution | Two-way ANOVA, Tukey post hoc | F (5, 54) = 11.10 | Sham vs. CCI <0.0001;  CCI vs. CCI+OE-RACK1:  D1 = 0.0704; D3, 7, 14 <0.0001;  CCI+OE-RACK1 vs. CCI+OE-RACK1+3-MA:  D1 = 0.7901; D3 = 0.001; D7 = 0.001; D14= 0.0019;  CCI+OE-RACK1 vs. CCI+OE-RACK1+Si-Beclin1:  D1 = 0.4840; D3 = 0.0287; D7 = 0.0287; D14 = 0.0456;  CCI+OE-RACK1 vs. CCI+OE-RACK1+Si-Atg5:  D1 = 0.6952; D3 = 0.0287; D7 = 0.006; D14 = 0.0287; |
| 5B | 10 | Normal  distribution | Two-way ANOVA, Tukey post hoc | F (5, 54) = 50.15 | Sham vs. CCI <0.0001;  CCI vs. CCI+OE-RACK1:  D1 = 0.0798; D3, 7, 14 <0.0001;  CCI+OE-RACK1 vs. CCI+OE-RACK1+3-MA:  D1 >0.9999; D3, 7, 14 <0.0001;  CCI+OE-RACK1 vs. CCI+OE-RACK1+Si-Beclin1:  D1 = 0.6325; D3 <0.0001; D7 = 0.0001; D14 <0.0001;  CCI+OE-RACK1 vs. CCI+OE-RACK1+Si-Atg5:  D1 = 0.3301; D3 = 0.0010; D7 = 0.0343; D14 = 0.0193; |
| 5C | 10 | Normal  distribution | Two-way ANOVA, Tukey post hoc | F (5, 54) = 19.63 | Sham vs. CCI <0.0001;  CCI vs. CCI+OE-RACK1:  D1 = 0.0765; D3, 7, 14 <0.0001;  CCI+OE-RACK1 vs. CCI+OE-RACK1+3-MA:  D1 = 0.6443; D3, 7, 14 <0.0001;  CCI+OE-RACK1 vs. CCI+OE-RACK1+Si-Beclin1:  D1 = 0.7217; D3 = 0.0026; D7 <0.0001; D14 = 0.001;  CCI+OE-RACK1 vs. CCI+OE-RACK1+Si-Atg5:  D1 = 0.8979; D3 = 0.0135; D7 = 0.0023; D14 = 0.0411; |
| 5E | 10 | Normal  distribution | Two-way ANOVA, Tukey post hoc | F (5, 54) = 5.621 | Sham vs. CCI <0.0001;  CCI vs. CCI+OE-RACK1:  D17 = 0.0678; D18, 19, 20 <0.0001;  CCI+OE-RACK1 vs. CCI+OE-RACK1+3-MA:  D17 = 0.4749; D18 = 0.0035;D19 = 0.0006; D20 <0.0001;  CCI+OE-RACK1 vs. CCI+OE-RACK1+Si-Beclin1:  D17 = 0.8720; D18 = 0.0409; D19 <0.0001; D20 <0.0001;  CCI+OE-RACK1 vs. CCI+OE-RACK1+Si-Atg5:  D17 = 0.5025; D18 = 0.0283; D19 = 0.0023; D20 = 0.0045; |
| 5F | 10 | Normal  distribution | One-way ANOVA, Tukey post hoc | F (5, 54) = 38.19 | Sham vs. CCI <0.0001;  CCI vs. CCI+OE-RACK1<0.0001;  CCI+OE-RACK1 vs. CCI+OE-RACK1+3-MA <0.0001;  CCI+OE-RACK1 vs. CCI+OE-RACK1+Si-Beclin1 <0.0001;  CCI+OE-RACK1 vs. CCI+OE-RACK1+Si-Atg5 = 0.0012; |
| 5G | 10 | Non-normal  distribution | Kruskal-Wallis test, Dunn post hoc |  | Sham vs. CCI = 0.0015;  CCI vs. CCI+OE-RACK1 = 0.0128;  CCI+OE-RACK1 vs. CCI+OE-RACK1+3-MA = 0.0364;  CCI+OE-RACK1 vs. CCI+OE-RACK1+Si-Beclin1 0.0013;  CCI+OE-RACK1 vs. CCI+OE-RACK1+Si-Atg5 = 0.0380; |
| 5H | 10 | Normal  distribution | One-way ANOVA, Tukey post hoc | F (5, 54) = 0.5083 | Sham vs. CCI = 0.9591;  CCI vs. CCI+OE-RACK1 = 0.9879;  CCI+OE-RACK1 vs. CCI+OE-RACK1+3-MA = 0.9823;  CCI+OE-RACK1 vs. CCI+OE-RACK1+Si-Beclin1 = 0.9906;  CCI+OE-RACK1 vs. CCI+OE-RACK1+Si-Atg5 = 0.9994; |
| 5J | 6 | Normal  distribution | One-way ANOVA, Tukey post hoc | F (4, 25) = 10.12 | CCI vs. CCI+OE-RACK1 <0.0001;  CCI+OE-RACK1 vs. CCI+OE-RACK1+3-MA = 0.0004;  CCI+OE-RACK1 vs. CCI+OE-RACK1+Si-Beclin1 = 0.0282;  CCI+OE-RACK1 vs. CCI+OE-RACK1+Si-Atg5 = 0.0162; |
| 6B | 6 | Normal  distribution | One-way ANOVA, Tukey post hoc | F (5, 30) = 27.34 | Sham vs. CCI = 0.0046;  CCI+Si-Ctrl vs. CCI+Si-RACK1 = 0.0062;  CCI+OE-Ctrl vs. CCI+OE-RACK1 = 0.0009; |
| 6F | 6 | Normal  distribution | One-way ANOVA, Tukey post hoc | F (5, 30) = 35.59 | Sham vs. CCI = 0.0017;  CCI+Si-Ctrl vs. CCI+Si-RACK1 <0.0001;  CCI+OE-Ctrl vs. CCI+OE-RACK1 <0.0001; |
| 6G | 6 | Normal  distribution | One-way ANOVA, Tukey post hoc | F (5, 30) = 29.90 | Sham vs. CCI = 0.0006;  CCI+Si-Ctrl vs. CCI+Si-RACK1 = 0.0057;  CCI+OE-Ctrl vs. CCI+OE-RACK1 <0.0001; |
| 7B | 6 | Normal  distribution | One-way ANOVA, Tukey post hoc | F (4, 25) = 13.77 | CCI+OE-RACK1+Veh vs. CCI+OE-RACK1+GSK = 0.0009 |
| 7C | 6 | Normal  distribution | One-way ANOVA, Tukey post hoc | F (4, 25) = 18.99 | CCI+OE-RACK1+Veh vs. CCI+OE-RACK1+GSK = 0.0004;  CCI+OE-RACK1+Si-Ctrl vs. CCI+OE-RACK1+Si-ATF4 = 0.0002; |
| 7D.LC3 | 6 | Normal  distribution | One-way ANOVA, Tukey post hoc | F (4, 25) = 12.26 | CCI+OE-RACK1+Veh vs. CCI+OE-RACK1+GSK = 0.0022;  CCI+OE-RACK1+Si-Ctrl vs. CCI+OE-RACK1+Si-ATF4 = 0.0022; |
| 7D.Beclin1 | 6 | Normal  distribution | One-way ANOVA, Tukey post hoc | F (4, 25) = 7.912 | CCI+OE-RACK1+Veh vs. CCI+OE-RACK1+GSK = 0.0259;  CCI+OE-RACK1+Si-Ctrl vs. CCI+OE-RACK1+Si-ATF4 = 0.043; |
| 7D.Atg5 | 6 | Normal  distribution | One-way ANOVA, Tukey post hoc | F (4, 25) = 16.45 | CCI+OE-RACK1+Veh vs. CCI+OE-RACK1+GSK = 0.0002;  CCI+OE-RACK1+Si-Ctrl vs. CCI+OE-RACK1+Si-ATF4 = 0.0024; |
| 7F | 6 | Normal  distribution | One-way ANOVA, Tukey post hoc | F (4, 25) = 10.54 | CCI+OE-RACK1+Veh vs. CCI+OE-RACK1+GSK = 0.0108;  CCI+OE-RACK1+Si-Ctrl vs. CCI+OE-RACK1+Si-ATF4 = 0.0041; |
| 7G | 6 | Normal  distribution | One-way ANOVA, Tukey post hoc | F (4, 25) = 15.15 | CCI+OE-RACK1+Veh vs. CCI+OE-RACK1+GSK = 0.0008;  CCI+OE-RACK1+Si-Ctrl vs. CCI+OE-RACK1+Si-ATF4 = 0.0017; |
| 7H | 6 | Normal  distribution | One-way ANOVA, Tukey post hoc | F (4, 25) = 16.40 | CCI+OE-RACK1+Veh vs. CCI+OE-RACK1+GSK = 0.0111;  CCI+OE-RACK1+Si-Ctrl vs. CCI+OE-RACK1+Si-ATF4 = 0.0002; |
| 7I | 6 | Normal  distribution | One-way ANOVA, Tukey post hoc | F (4, 25) = 17.40 | CCI+OE-RACK1+Veh vs. CCI+OE-RACK1+GSK <0.0001;  CCI+OE-RACK1+Si-Ctrl vs. CCI+OE-RACK1+Si-ATF4 = 0.0001; |
| 7K | 6 | Normal  distribution | One-way ANOVA, Tukey post hoc | F (4, 25) = 14.18 | CCI+OE-RACK1+Veh vs. CCI+OE-RACK1+GSK = 0.0001;  CCI+OE-RACK1+Si-Ctrl vs. CCI+OE-RACK1+Si-ATF4 = 0.0008; |
